# Researchers’ experience in data collection with families of femicide victims

**DOI:** 10.1590/0034-7167-2023-0119

**Published:** 2024-09-20

**Authors:** Igor de Oliveira Reis, Edson Arthur Scherer, Zeyne Alves Pires Scherer

**Affiliations:** I Universidade de São Paulo. Ribeirão Preto, São Paulo, Brazil

**Keywords:** Qualitative Research, Interview, Family Relations, Gender-Based Violence, Femicide, Investigación Cualitativa, Entrevista, Relaciones Familiares, Violencia de Género, Feminicidio

## Abstract

**Objectives:**

to describe researchers’ experience in collecting data from families of femicide victims.

**Methods:**

this descriptive, qualitative study took the form of an experience report and was conducted in Manaus, Amazonas, Brazil. It involved documentary consultation, training researchers, scheduling and conducting interviews, and using a field diary to record the researchers’ perceptions and experiences.

**Results:**

the descriptions and photographs of the crime scene were both distressing and impactful for the researchers. The mementos of the victims (including clothing, objects, and childhood photos) shown by their families were deeply moving. Identifying with these experiences facilitated listening to the stories told by the relatives. It was essential to maintain a non-judgmental attitude, acknowledge the loss, provide support for the suffering, and demonstrate a willingness to help.

**Final Considerations:**

the experience encompassed both theoretical and methodological aspects that were planned and executed in data collection, fostering the development of skills and sensitivity towards the cases. Beyond knowledge and preparation, researchers are expected to exhibit ethical conduct and empathetic capacity.

## RESUMEN

**Objetivos:**

describir la experiencia de los investigadores en la recopilación de datos con familias de víctimas de feminicidio.

**Métodos:**

estudio descriptivo, cualitativo, tipo relato de experiencia, realizado en Manaos, Amazonas, Brasil. Se llevó a cabo una consulta documental, capacitación de los investigadores, programación y realización de entrevistas, utilizando un diario de campo para el registro de las percepciones y vivencias de los investigadores.

**Resultados:**

la descripción y las fotografías de la escena del crimen afectaron e impactaron a los investigadores. Los recuerdos de la víctima (ropa, objetos y fotos de la infancia), mostrados por la familia, los conmovieron. Identificar estas vivencias facilitó escuchar la historia relatada por los familiares. Mantener una actitud de no juicio, no negación de la pérdida, acogida del sufrimiento y demostración de disponibilidad para ayudar fueron fundamentales.

**Consideraciones Finales:**

la experiencia abarcó aspectos teórico-metodológicos planificados y ejecutados en la recopilación de datos, proporcionando el desarrollo de habilidades y la sensibilización con los casos. Además del conocimiento y preparación, se espera una actitud ética y capacidad empática de los investigadores.

## INTRODUCTION

The use of interviews as a data collection technique in qualitative research, aimed at exploring social phenomena, has become increasingly prevalent. This technique collects individual or group information about specific problems under investigation, encompassing a range of perceptions, knowledge, ideas, opinions, beliefs, feelings, experiences, life events, and behaviors. It enables closer engagement with the subjective aspects of the subjects^(1)^.

Conducting these studies requires researchers to carefully consider the method, emphasizing data collection instruments, as well as the recruitment, acceptance, and participation of participants^(2)^. Depending on the research subject, it can become a challenging process, especially in themes related to complex phenomena such as femicide.

Femicide, considered the ultimate form of gender-based violence against women, occurs within contexts of domestic and family violence, contempt, or discrimination against the female condition^(3)^. A systematic literature review^(4)^ identified factors related to intimate partner femicide. Among perpetrators, motivators included being a victim of violence in childhood, the presence of mental disorders, jealousy, sexual issues, and access to weapons. For victims, insecurity, low education, and financial and/or emotional dependence were significant. Factors associated with both aggressors and victims included age differences, repeated violence, cohabitation, separation/divorce, and stalking. Rural areas were noted as the most common settings for such occurrences.

Beyond the direct female victim of femicide, other family members are part of the scenario and are considered indirect victims. As a primary and natural institution, the family generally assumes a protective role and tends to experience intensely the loss of one of its members. In cases of femicide, children are left motherless, with fathers imprisoned or fugitive. Other relatives, such as siblings and nephews, may encounter socioeconomic challenges, as well as shortand long-term physical, mental, sexual, and reproductive health issues. These situations can further extend and complicate the grieving process^(5-6)^.

In Brazil, studies^(5-8)^ with families of femicide victims described the data collection procedures adopted by researchers and the difficulties encountered. All employed interviews for data collection and identified as crucial elements for their conduct: the identification of relatives through local records, the interviewers’ prior experience, the need for a respectful approach, and adherence to ethical aspects. In one study^(8)^, where a nurse conducted interviews, the intermediation of another professional in approaching the interviewees was suggested. The most notable difficulties were non-response or failure to return contact attempts with the families. The reasons, according to the researchers, included discomfort in discussing the situation due to ongoing fear of the aggressor and reluctance to discuss the experienced violence, to avoid recalling the loss. Additionally, some relatives refused to have the interviews recorded.

Therefore, investigating femicide requires care, as the topic is complex and involves all parties, including the researchers. When addressing families who have experienced the loss of a member to femicide, a space is provided for interviewees to speak, be heard, reflect, and share their suffering. The researchers, in hearing these stories, undergo an experience that, in addition to producing knowledge, can mobilize feelings and interests in providing assistance.

## OBJECTIVES

To describe researchers’ experiences in data collection with families of femicide victims.

## METHODS

This text describes a qualitative, descriptive study in the form of an experience report, part of the data collection phase of the master’s thesis titled “Violence and Femicide: Social Representations of Femicide Victims’ Families”. The research was approved by the Research Ethics Committee of the Ribeirão Preto College of Nursing at the University of São Paulo (EERP/USP).

The method used was a field diary, compiled by researchers, with entries that included their perceptions and experiences during various stages of the research: reviewing documents of femicide cases at the Legal Medical Institute (IML) in Manaus, training the researchers, contacting families for scheduling and conducting semi-structured interviews^(1)^.

The documentary consultation of femicide cases recorded between 2018 and 2020 took place from July to November 2021, resulting in the inclusion of 151 occurrences, categorized as suspected and confirmed. The families were identified by name, phone number, and address, listed in the Death Recognition Declaration (DRO) and the Cadaver Removal and Release Guide.

The two researchers responsible for data collection were nurses, specialists in psychiatric nursing and mental health, members of the Interdisciplinary Study Group on Violence (GREIVI) at EERP/USP. They had clinical and scientific experience with violence issues, as well as in conducting qualitative interviews. Additionally, they underwent training in family approach and therapeutic communication, with realistic simulation, offered by the psychosocial sector of the IML, in December 2021.

After training, telephone calls were made to the families. Contacts followed the sequence from the oldest to the most recent cases, and from confirmed to suspected ones. The following order of communication was established: 1) confirmation of the family member’s name (as identified in the documents); 2) introduction of the researcher (name, profession, and origin); 3) explanation of the reasons for the call (how the family member was identified, confirmation of the case and the connection with the victim); 4) explanation of the importance of participating in the research; 5) invitation for a face-to-face conversation at the time, date, and place of the family member’s preference. If the family member was unavailable, another close family member of the victim was requested. In case of refusal or inability to contact, the next registered family member was sought.

With the initiation of phone contacts, difficulties emerged. In 76 of the 151 selected femicide cases, the phone numbers in the records were unavailable or nonexistent, making location impossible. Another 20 declined to participate in the interview, citing no interest in speaking about the subject due to it reminding them of suffering or because the perpetrator was already imprisoned. However, in the 7 interviews conducted, data saturation was observed, allowing an understanding of the phenomenon under study^(1-2)^. Therefore, it was not necessary to contact the remaining 48 families.

The interviews were conducted in January and February 2022, in a private room at the IML or at the families’ residences, as per their preference. The interviewees, a father, a mother, a daughter, two sisters, an uncle, and a cousin, had significant ties to the victim. At the end of each interview, a Public Services Guide (an intersectoral network of health, psychosocial, police, and legal support services) was provided to offer information and facilitate access to services, often unknown to the families. Written informed consent was obtained from all individuals involved in the study.

## RESULTS

Access to the families of femicide victims in this study was facilitated through the review of documents at the Legal Medical Institute (IML), where all violent deaths in the city are registered. The request for access to these cases and family information was made to the Public Security Department of Amazonas (SSP/AM), which then forwarded the request to the Department of Technical-Scientific Police (DPTC), and subsequently to the IML. The process of obtaining authorization for the study involved multiple meetings with the management of both the DPTC and IML, proving to be a challenging stage for the researchers due to difficulties in contacting the relevant authorities.

During the document review, the researchers encountered shocking photographs of the crime scenes, including images of victims’ bodies in states of bloodiness or advanced decomposition. These images caused distress, particularly as they detailed the violent circumstances of the deaths. Most deaths occurred in the victims’ homes, primarily caused by gunshot wounds or the use of bladed weapons, as well as other methods such as physical assault, strangulation, suffocation, drowning, and poisoning. In some instances, the bodies were concealed by the perpetrators.

Despite being familiar with the theme of violence, the researchers experienced discomfort and revulsion upon seeing these records, becoming increasingly sensitized to the pain and suffering of the victims’ families. This exposure had a profound impact on them, raising concerns about how to approach the families without retriggering their traumatic experiences.

In this context, the training at the IML was extremely valuable, as it integrated the theoretical instructions discussed in GREIVI with institutional practices. Conducted in person after the document review, over two weekends, the training consisted of four sessions. The psychologist and social worker from the department covered general aspects of family approaches, focusing on respect for dignity and human rights, confidentiality of information, privacy protection, and ensuring that no additional harm was caused, along with guidelines for specific cases. Techniques in therapeutic communication were taught, emphasizing the ability to clearly explain the sequence of events. The importance of building trust with the families, which may require time and effort, was also underscored.

As a practical component of the training, the researchers, under the supervision of the institution’s psychosocial sector professionals, made phone calls to families of victims of violent deaths handled that day. They informed the families about the incidents and invited them to the IML, where they were received in person for document signing and explanations of the post-mortem processes. The researchers accompanied the families during the victim identification procedure, revealing the identity and relationship ascertained from fingerprint and DNA examinations. Additionally, they offered emotional support to the victims and families of domestic and sexual violence present for forensic examinations.

After this phase, the researchers began contacting the families of femicide victims. The priority was to schedule the interviews as soon as possible to ensure the availability of the interviewees. However, it was noted that out of every 15 calls made, only one family member agreed to participate in the interview, a situation justified by the sensitive nature of the subject matter.

Regarding the location chosen by the study participants for the interviews, four opted for the Legal Medical Institute (IML), as they did not feel comfortable inviting strangers into their homes. Conversely, due to the negative associations with the IML, three other families preferred to have the interviews conducted in their homes. During these home interviews, some family members displayed rooms, including the victim’s bedroom, with personal items such as clothes, belongings, and childhood photos, deeply affecting the researchers.

The strategies employed in contacting the families, especially during the interviews, included: listening attentively to their stories, using clear and accessible language while avoiding jargon and interruptions; refraining from judgment and showing sensitivity to cultural and ideological differences; acknowledging the loss, validating the accounts without making assumptions; offering support in the face of suffering by providing a comforting presence, maintaining eye contact, extending hands, and giving hugs; and demonstrating a willingness to help by providing additional material and advising them to seek professional assistance.

Despite recalling unpleasant events, such as incidents of violence against the victim, tumultuous interactions with the aggressor, receiving news of the femicide, and the circumstances of its occurrence, the researchers observed that the family members were able to discuss their loss. To facilitate a comfortable environment for sharing these experiences, it was essential to legitimize the feelings expressed by the family members in both words and behaviors (Chart 1).

**Chart 1:** Phrases used by the researchers to legitimize feelings, as expressed by the family members during the interviews, Manaus, Amazonas, Brazil, 2022

Expressed Feelings	Legitimization Phrases
Shame	Feel comfortable here.
Incomprehension	I understand you.
Doubt	I believe you.
Sadness	I am sorry for what happened to you. I am sorry for what happened to her. I am sorry that you are going through this.
Guilt	You did what was within your power.
Longing	I can imagine how important she was to you. I can imagine how much you loved her.
Helplessness	When you’re ready, you will be able to [move forward/cope/manage].

Another approach that strengthened interpersonal relationships and facilitated detailed discourse with the families was the use of therapeutic communication, both in phone calls and during interviews. Techniques such as expressing interest and acceptance, echoing or repeating the interviewee’s last words to encourage further dialogue, and using moments of silence to allow for emotional responses, like brief crying, or to encourage reflection for formulating responses were effectively employed.

It was observed that the family members were not receiving care or support from health and psychosocial sectors. One researcher experienced feelings of powerlessness and disbelief upon recognizing this lack of assistance. However, the phone calls and invitations to interviews were received positively by the families, as they had previously only been approached regarding legal matters. They expressed gratitude for the space provided, which in turn made the researchers feel gratified and confident that they had achieved their proposed objective.

As outlined in the methodology, a Public Services Guide was distributed at the end of each interview. Throughout their interactions with the participants, the researchers did not identify a need to recommend professional care or intervention from the services listed in the provided Guide.

## DISCUSSION

Investigating cases involving the families of femicide victims relies on the cooperation of researchers with public security institutions that possess data on such incidents, documents considered confidential. Although femicide is recognized in Brazilian legislation^(3)^, currently, there is no specific category in the Mortality Information System (SIM), produced by the health system, that provides data about this crime or the victims’ families. Consequently, researchers have been utilizing police records and judicial proceedings from the databases of the Civil Police and Public Prosecutor’s Office^(5-6,8)^, and cross-referencing data on female homicides from the SIM with prior notifications of interpersonal violence against women from the Notifiable Diseases Information System, focusing on death certificates (part III - place of residence, to locate the family)^(7)^. In this study, the hierarchy of state public agencies (SSP/AM, DPTC, and IML) was followed to access the Death Recognition Declaration (DRO) and the Cadaver Release Guide, documents through which the families were identified.

Another critical aspect is the training of researchers involved in approaching and interviewing these families. In a study conducted in a southeastern Brazilian metropolis^(6)^, researchers were trained in verbal autopsy to interview families of femicide victims. This technique, which investigates the causes and circumstances of death, is conducted shortly after the event. In this experience report, at least one year passed between the occurrence of the femicide and the interviews, aiming to understand the impact of femicide on the families’ lives and providing them with a space to share their experiences.

In this setting, researchers may encounter a range of emotions and reactions due to the impactful nature of the subject^(9)^. In qualitative research, the interviewer is not exempt from forming a relationship with the participant and is expected to do so with commitment and ethics. It is essential for the interviewer to be prepared to manage any sensitization that might arise, taking into account the profile of the participants, the context and timeframe they are in, and the established proximal processes^(2)^.

Researchers who interviewed health and social assistance professionals aiding families of homicide victims in their routine work reported difficulties in conducting the interviews, facing refusals to participate and to record the sessions. Feelings of uncertainty, apprehension, fear, and insecurity were prevalent among the professionals during their interactions with the families^(9)^. These feelings were similar to those experienced by the researchers of this study when reviewing the femicide case documents, but were mitigated by the training conducted at the IML before the interviews.

In addition to the training and qualifications of researchers, Minayo^(1-2)^ proposes criteria for data collection in qualitative research that were corroborated by the researchers in this study. These criteria include:

1. Reflexivity: This refers to the researchers’ own experiences, opinions, biases, and values, and examines how these aspects may influence the research, especially when investigating violence;
2. Preparation for the Field: This involves a literature review, a clear definition of the research objectives, and the development of a work plan;
3. Ethics and Cultural Sensitivity: This entails obtaining informed consent from participants, ensuring confidentiality, and respecting the cultural opinions and values of the interviewees;
4. Observation: This enables the researcher to adapt to and understand the main issues that may affect data quality;
5. Records: This involves documenting the learning and events throughout the research process in a careful and systematic manner;
6. Self-evaluation: This includes reflecting on the progress of the research, the challenges encountered, and the influence of the researcher’s own perspectives on data interpretation.

Regarding the modality of the interviews, face-to-face meetings offer greater credibility and reliability compared to online interviews. This approach enables the collection of detailed information and reveals the intersubjective aspects of the interviewees^(1-2)^. The emotional load involved is substantial, and the researchers were prepared to accommodate the participants while maintaining an investigative stance, thus avoiding transforming the situation into a counseling session. Should the need for professional care or intervention be identified, appropriate services would be indicated, as described in the Public Services Guide provided.

The residence has been a commonly used space to discuss delicate matters such as femicide^(5-8)^. Although some participants preferred the IML, it was observed that families felt comfortable in their chosen environments, which were safe, private, and ensured confidentiality.

The semi-structured interview script facilitated a cathartic and reflective experience for the families. Legitimizing the feelings and thoughts expressed by the interviewees was essential for establishing a bond with the researchers. By respecting the experiences and emotions of the families, and when a legitimate relationship and a safe space for conversation were perceived, they felt more confident to share and collaborate without fear of judgment^(10)^.

Legitimizing the family members’ speech involved showing consideration and respect for their opinions, experiences, and perspectives. Other forms of legitimization used included: listening attentively, validating feelings and experiences, asking clarifying questions, respecting diverse perspectives, avoiding premature judgments, respectfully presenting counterpoints, acknowledging the participant’s expertise, demonstrating genuine interest, supporting and encouraging expression, and fostering an inclusive environment^(10)^.

Upon encountering cases of femicide, especially when accessing information, images, and family members’ accounts about both the incident itself and its impacts on the family unit, researchers may be overwhelmed by feelings and perceptions of emotion and compassion. As humans, dealing with or being confronted with a crime of this nature, most often committed by someone close to the victim, can evoke discomfort and revulsion. In this study, this occurred. The challenge lies in not allowing oneself to be influenced or overwhelmed by these experiences, nor in denying them, but rather in identifying them. Therefore, feeling moved, in the experience of the researchers, ultimately facilitated a closer connection with the interviewees and their participation.

### Study limitations

This study has several limitations that should be acknowledged. The difficulty in contacting family members by phone, due to unavailability or lack of records, and their refusal to participate because they did not feel ready to discuss their grief experience, or because they considered the situation resolved in cases where the perpetrator was convicted and imprisoned, should be taken into account. Additionally, the sensitive nature of the topic may have influenced the participants’ willingness to share deeply personal and traumatic experiences. Another significant limitation is the potential emotional influence of the researchers on the collected data, despite efforts to maintain objectivity. The limited scientific literature on the topic hindered a more in-depth discussion based on existing research.

### Contributions to the Field

The approach outlined in this experience report for interviewing families of femicide victims can provide students, educators, health professionals, and researchers, specifically nurses, with strategies that can be reflected in their practices. By engaging in care or scientific activities of this nature, they can provide therapeutic value to families experiencing the repercussions of femicide. Participation in such investigations also allows for the intersection of the profession with other social themes, such as violence and gender.

## FINAL CONSIDERATIONS

The experience encompassed theoretical and methodological aspects planned and executed in qualitative data collection by the researchers. By undergoing this process, they developed skills and sensitivity to the cases and in interactions with the interviewed families.

The research group meetings (GREIVI), knowledge obtained from the literature, and the researchers’ experience in qualitative interviews, combined with the training received from the IML, were crucial in approaching the families and discussing a topic so significant in their lives. Additionally, these experiences served as a foundation for the researchers to direct the communication order and use appropriate language, both in phone calls and face-to-face interviews.

Conducting research with families of femicide victims is a challenging and sensitive process. Researchers need to demonstrate availability and respect for greater interaction with participants, ensuring quality data collection. Therefore, in addition to knowledge and preparation, an ethical attitude is expected, without sacrificing empathic capacity towards the study’s target population. On the other hand, it is recommended to improve public policies for supporting the families of femicide victims. Such policies, with the aid of psychological, social, and legal assistance, could help in processing grief and overcoming the consequences of experienced violence.
