# Supplementary material for: Researchers’ experience in data collection with families of femicide victims
Source: Rev Bras Enferm. 2024 Sep 20;77(4):e20230119. doi: 10.1590/0034-7167-2023-0119 (PMC11419681; doi:10.1590/0034-7167-2023-0119)
Supplement: Translation

# Translation: Experiência de pesquisadores na coleta de dados com famílias de vítimas de feminicídio

**DOI:** 10.1590/0034-7167-2023-0119pt

**Authors:** Igor de Oliveira Reis, Edson Arthur Scherer, Zeyne Alves Pires Scherer

**Affiliations:** I Universidade de São Paulo. Ribeirão Preto, São Paulo, Brasil

**Keywords:** Pesquisa Qualitativa, Entrevista, Relações Familiares, Violência de Gênero, Feminicídio

## Abstract

**Objetivos:**

descrever a experiência de pesquisadores na coleta de dados com famílias de vítimas de feminicídio.

**Métodos:**

estudo descritivo, qualitativo, tipo relato de experiência, realizado em Manaus, Amazonas, Brasil. Foi realizada uma consulta documental, capacitação dos pesquisadores, agendamento e realização de entrevistas, utilizando um diário de campo para o registro das percepções e vivências dos pesquisadores.

**Resultados:**

a descrição e as fotografias da cena do crime afligiram e impactaram os pesquisadores. As lembranças da vítima (roupas, objetos e fotos de infância), mostradas pela família, os comoveram. Identificar essas vivências facilitou ouvir a história relatada pelos familiares. Manter uma atitude de não julgamento, não negação da perda, acolhimento do sofrimento e demonstração de disponibilidade para ajudar foram fundamentais.

**Considerações Finais:**

a experiência perpassou aspectos teóricos-metodológicos planejados e executados na coleta de dados, proporcionando o desenvolvimento de habilidades e a sensibilização com os casos. Além de conhecimento e preparo, espera-se atitude ética e capacidade empática dos pesquisadores.

## INTRODUÇÃO

A entrevista como técnica de coleta de dados em pesquisas com abordagens qualitativas tem sido cada vez mais utilizada com o objetivo de explorar fenômenos sociais. Essa técnica obtém informações individuais ou em grupo sobre determinado problema a ser investigado, contemplando o conjunto de percepções, conhecimentos, ideias, opiniões, crenças, sentimentos, experiências, vivências e comportamentos, permitindo uma aproximação com os aspectos subjetivos dos sujeitos^(1)^.

A condução desses estudos requer do pesquisador um olhar cuidadoso para o método, salientando os instrumentos de coleta de dados, bem como o recrutamento dos participantes, sua aceitação e participação^(2)^. Dependendo do objeto de estudo, pode se tornar um processo desafiador, principalmente em temas relacionados a fenômenos complexos como o feminicídio.

Considerado a última instância da violência de gênero contra a mulher, o feminicídio está inserido nos contextos de violência doméstica e familiar, menosprezo ou discriminação à condição de ser mulher^(3)^. Um estudo de revisão sistemática da literatura^(4)^ elencou fatores relacionados ao feminicídio por parceiro íntimo. Entre os agressores, foram listados motivadores como ter sido vítima de violência na infância, a presença de transtornos mentais, ciúmes, problemas sexuais, a compra e o acesso a armas. Nas vítimas, a insegurança, a baixa escolaridade e a dependência financeira e/ou emocional foram aspectos apontados. Como fatores associados a agressores e a vítimas figuraram a diferença de idade, a violência repetida, o convívio no mesmo domicílio, a separação/divórcio e a perseguição. No que se refere ao ambiente, as áreas rurais foram o local com mais ocorrências.

Além da mulher, vítima direta do feminicídio, outros integrantes da família compõem o cenário e são considerados vítimas indiretas. Por ser uma instituição primária e natural, em geral, a família é remetida a um papel protetivo e tende a vivenciar com intensidade a perda de um de seus componentes. Na ocorrência de feminicídio, crianças ficam órfãs de mães e com pais presos ou foragidos. Outros familiares, como irmãos e sobrinhos, podem enfrentar dificuldades socioeconômicas, além de problemas em suas saúdes física, mental, sexual e reprodutiva a curto e longo prazo. Essas situações podem tornar ainda mais longo e difícil o processo do luto^(5-6)^.

No Brasil, estudos^(5-8)^ com famílias de vítimas de feminicídio descreveram os procedimentos adotados pelos pesquisadores na coleta de dados com os participantes e as dificuldades que encontraram. Todos utilizaram a entrevista para coleta e consideraram como elementos fundamentais para desenvolvê-las: a identificação dos familiares por meio de registros locais; a experiência prévia dos entrevistadores; a necessidade de uma abordagem respeitosa; e o cumprimento dos aspectos éticos. Em uma dessas pesquisas^(8)^, em que a condução das entrevistas foi feita por um enfermeiro, foi também sugerida a intermediação de outro profissional na aproximação com os entrevistados. Entre as dificuldades encontradas, destacaram-se o não atendimento ou o não retorno às tentativas de contato com as famílias. As justificativas, segundo os pesquisadores, foram o desconforto em falar da situação por ainda estarem com medo do agressor e a proibição em discorrer sobre a violência vivenciada, como forma de evitar a lembrança da perda. Além disso, alguns familiares não aceitaram que as entrevistas fossem gravadas.

Portanto, investigar o feminicídio requer cuidados, pois o tema é complexo e mobiliza todos os envolvidos, inclusive os pesquisadores. Ao abordar famílias que vivenciaram a perda de uma integrante por feminicídio com o propósito de investigar essa situação e suas repercussões, é oferecido um espaço para os entrevistados falarem, serem ouvidos, refletirem e compartilharem seu sofrimento. Os pesquisadores, por sua vez, ao ouvirem os relatos, vivenciam uma experiência que, além de produzir conhecimento, pode mobilizar sentimentos e interesses em ajudar.

## OBJETIVOS

Descrever a experiência de pesquisadores na coleta de dados com famílias de vítimas de feminicídio.

## MÉTODOS

Este texto descreve um estudo descritivo qualitativo do tipo relato de experiência, parte da etapa de coleta de dados da dissertação de mestrado intitulada “Violência e feminicídio: representações sociais de familiares das vítimas de feminicídio”. A pesquisa foi aprovada pelo Comitê de Ética em Pesquisa da Escola de Enfermagem de Ribeirão Preto da Universidade de São Paulo (EERP/USP).

O método utilizado foi o diário de campo, construído pelos pesquisadores, com registros que contemplaram suas percepções e vivências durante vários momentos da pesquisa: consulta aos documentos dos casos de feminicídios no Instituto Médico Legal (IML) de Manaus, capacitação dos pesquisadores, contato com os familiares para agendamento e realização das entrevistas semiestruturadas^(1)^.

A consulta documental dos casos de feminicídio registrados entre 2018 e 2020 ocorreu de julho a novembro de 2021, resultando na inclusão de 151 ocorrências, classificadas em suspeitas e confirmadas. Os familiares foram identificados por nome, telefone e endereço, constantes na Declaração de Reconhecimento de Óbito (DRO) e na Guia de Remoção e Liberação de Cadáver.

Os dois pesquisadores responsáveis pela coleta de dados eram enfermeiros, especialistas em enfermagem psiquiátrica e saúde mental, integrantes do Grupo de Estudos Interdisciplinar sobre Violência (GREIVI) da EERP/USP. Possuíam experiência assistencial e científica com a temática da violência, bem como na condução de entrevistas qualitativas. Complementarmente, passaram por uma capacitação de abordagem familiar e comunicação terapêutica, com simulação realística, oferecida pelo setor psicossocial do IML, em dezembro de 2021.

Após a capacitação, realizaram ligações telefônicas para os familiares. Os contatos seguiram a sequência dos casos mais antigos para os mais recentes, e dos confirmados para os suspeitos. Foi estabelecida a seguinte ordem de comunicação: 1) confirmação do nome do familiar (se era o mesmo identificado nos documentos); 2) apresentação do pesquisador (nome, profissão e procedência); 3) explicação dos motivos da ligação (como o familiar foi identificado, confirmação do caso e do vínculo com a vítima); 4) explanação sobre a importância da participação na pesquisa; 5) convocação para uma conversa presencial no dia, horário e local de preferência do familiar. Caso o familiar não estivesse disponível, solicitava-se a indicação de outro integrante da família próximo à vítima. Em caso de negação do convite ou impossibilidade de conseguir o contato, procurava-se o próximo familiar registrado.

Com o início dos contatos telefônicos, surgiram dificuldades. Em 76 dos 151 feminicídios selecionados, os números de telefone constantes nos registros estavam indisponíveis ou não existiam, impossibilitando a localização. Outros 20 não aceitaram participar da entrevista, justificando não ter interesse em falar sobre o assunto por remetê-los ao sofrimento ou porque o autor do crime já estava preso. No entanto, nas 7 entrevistas realizadas, observou-se a saturação dos dados, permitindo a compreensão do fenômeno em estudo^(1-2)^. Portanto, não foi necessário contatar os 48 familiares restantes.

As entrevistas foram realizadas nos meses de janeiro e fevereiro de 2022, em uma sala reservada no IML ou na residência dos familiares, conforme a preferência destes. Os entrevistados, um pai, uma mãe, uma filha, duas irmãs, um tio e uma prima, tinham importante vínculo com a vítima. Ao final de cada entrevista, entregava-se um Guia de Serviços Públicos (rede intersetorial de serviços de apoio de saúde, assistência psicossocial, policial e jurídica) com o objetivo de disponibilizar informações e facilitar o acesso dos familiares a serviços, por vezes desconhecidos. A assinatura do Termo de Consentimento Livre e Esclarecido foi obtida de todos os indivíduos envolvidos no estudo por escrito.

## RESULTADOS

O acesso aos familiares das vítimas de feminicídio no estudo foi viabilizado através da consulta a documentos no Instituto Médico Legal (IML), onde são registradas todas as mortes violentas da cidade. A solicitação para acessar os casos e as informações dos familiares foi feita à Secretaria de Segurança Pública do Amazonas (SSP/AM), que encaminhou a demanda para o Departamento de Polícia Técnico Científica (DPTC), e posteriormente para o IML. O processo de obtenção da autorização para a realização do estudo envolveu várias reuniões com a direção da DPTC e do IML, sendo uma etapa desgastante para os pesquisadores devido às dificuldades de contato com as instâncias relevantes.

Durante a consulta dos documentos, os pesquisadores se depararam com fotografias chocantes das cenas dos crimes, incluindo corpos das vítimas em estado ensanguentado ou de decomposição avançada. Estas imagens provocaram comoção, especialmente por detalhar as circunstâncias violentas das mortes. A maioria dos óbitos ocorreu nas residências das vítimas, causados principalmente por disparos de arma de fogo ou uso de arma branca, além de outros métodos como agressão física, estrangulamento, sufocamento, afogamento e envenenamento. Em alguns casos, os corpos foram ocultados pelos perpetradores.

Apesar da familiaridade com a temática da violência, os pesquisadores sentiram incômodo e repulsa diante dos registros, sensibilizando-se com a dor e o sofrimento dos familiares das vítimas. Essa exposição os impactou profundamente, levantando preocupações sobre como abordar os familiares sem reavivar suas experiências traumáticas.

Nesse contexto, a capacitação no IML foi extremamente importante, pois proporcionou a integração das instruções teóricas, discutidas no GREIVI, com as práticas institucionais. Realizada após a consulta documental, de forma presencial, durante dois finais de semana, totalizou quatro encontros. O psicólogo e a assistente social do setor explicaram aspectos gerais das abordagens às famílias, focando no respeito à dignidade e aos direitos humanos, na confidencialidade das informações, na proteção da privacidade e na garantia de que nenhum dano adicional fosse causado, além dos direcionamentos exigidos por casos específicos. Foram ensinadas técnicas de comunicação terapêutica, com ênfase na capacidade de explicar de forma compreensível a sequência dos fatos ocorridos. Também destacaram a importância da construção de confiança com os familiares, que pode exigir tempo e esforço.

Como parte prática das atividades de capacitação, os pesquisadores realizaram, sob supervisão dos profissionais do setor psicossocial da instituição, telefonemas a familiares de vítimas de mortes violentas atendidas naquele dia. Informaram as famílias sobre o ocorrido e as convocaram a comparecer ao IML, onde as acolheram presencialmente para a assinatura de documentos e explicaram os processos pós-morte. Acompanharam os familiares no procedimento de reconhecimento da vítima, revelando a identidade e o vínculo resultantes do exame papiloscópico e de DNA. Além disso, ofereceram suporte emocional às vítimas e aos familiares de violência doméstica e sexual presentes para exame de corpo de delito.

Após essa etapa, iniciou-se o contato com os familiares das vítimas de feminicídio. Priorizou-se realizar o agendamento das entrevistas o mais breve possível, para assegurar a presença dos entrevistados. Contudo, foi observado que a cada 15 ligações realizadas, apenas um familiar aceitava participar da entrevista, situação justificada pela delicadeza do assunto abordado.

Quanto ao local escolhido pelos participantes do estudo para a realização das entrevistas, quatro optaram pelo IML, por não se sentirem à vontade para receber desconhecidos em suas casas. Por outro lado, a representação negativa que o IML trazia para outros três familiares fez com que preferissem as entrevistas em seus domicílios. Nas entrevistas realizadas nas casas, alguns familiares mostraram cômodos, incluindo o quarto da vítima, com lembranças como roupas, objetos e fotos de infância, emocionando os pesquisadores.

As estratégias utilizadas em todo o processo de contato com os familiares, especialmente nas entrevistas, foram: ouvir atentamente a história, utilizando uma comunicação clara e linguagem acessível, evitando jargões e interrupções; não julgar, demonstrando sensibilidade para diferenças culturais e ideológicas; não negar a perda, validando os relatos e evitando suposições; acolher o sofrimento, transmitindo presença, fixando olhares, dispondo as mãos e oferecendo abraços; e demonstrar disponibilidade em ajudar, entregando material complementar e orientando-os a buscar assistência profissional.

Apesar das lembranças de fatos desagradáveis, como episódios de violência presenciados contra a vítima, a convivência conturbada com o agressor, a notícia do feminicídio e a maneira como ocorreu, os pesquisadores perceberam que os familiares conseguiram falar sobre a perda. Para fazer com que se sentissem à vontade em compartilhar essas situações, foi necessário legitimar os sentimentos potencialmente expressos por falas e comportamentos dos familiares (Quadro 1).

**Quadro 1:** Frases de legitimação dos sentimentos, utilizadas pelos pesquisadores, de acordo com o que foi expressos pelos familiares nas entrevistas, Manaus, Amazonas, Brasil, 2022

Sentimentos expressos	Frases de legitimação
Vergonha	Sinta-se à vontade aqui
Incompreensão	Eu entendo você
Dúvida	Eu acredito em você
Tristeza	Eu sinto muito pelo que aconteceu com você Eu sinto muito pelo que aconteceu com ela Eu sinto muito que esteja passando por isso
Culpa	Você fez o que estava no seu alcance
Saudade	Eu imagino o quanto ela era importante pra você Eu imagino o quanto você a amava
Incapacidade	Quando puder, você vai conseguir

Outra abordagem que fortaleceu o relacionamento interpessoal e favoreceu o discurso detalhado dos familiares foi a utilização da comunicação terapêutica, tanto nas ligações telefônicas quanto nas entrevistas. A verbalização de interesse e aceitação, a reafirmação ou repetição das últimas palavras do entrevistado como estímulo para que continuasse sua fala, o uso de instantes de silêncio para permitir respostas emocionais como choro breve, ou para propiciar reflexões que permitissem a formulação de respostas, foram estratégias utilizadas.

Foi observado que os familiares não estavam sendo cuidados ou mesmo recebendo suporte dos setores de saúde e psicossocial. Para um dos pesquisadores, o sentimento suscitado foi de impotência e descrédito ao perceber essa desassistência. Por outro lado, a ligação telefônica e a proposta de entrevista foram referidas como positivas pelos familiares, pois até então só tinham sido procurados para tratar questões jurídicas. Eles mostraram gratidão pelo espaço oferecido, o que fez os pesquisadores se sentirem gratificados e com a sensação de terem alcançado o objetivo proposto.

Como descrito no método, foi entregue, ao final de cada entrevista, um Guia de Serviços Públicos. Os pesquisadores, ao longo do contato com os participantes, não detectaram a necessidade de indicar atendimento ou intervenção profissional dos serviços constantes no Guia disponibilizado.

## DISCUSSÃO

A realização de investigações com familiares de vítimas de feminicídio depende da articulação dos pesquisadores com as instituições de segurança pública que detêm os dados das ocorrências, documentos considerados sigilosos. Apesar de o feminicídio constar na legislação brasileira^(3)^, não há, até o momento, uma categoria no Sistema de Informações sobre Mortalidade (SIM), produzido pelo sistema de saúde, que disponibilize informações sobre esse crime em seus dados, tampouco sobre os familiares das vítimas. Por isso, pesquisadores têm utilizado registros policiais e processos judiciais do banco de dados da Polícia Civil e do Ministério Público^(5-6,8)^, e o cruzamento dos dados de homicídios femininos do SIM com as notificações prévias de violência interpessoal contra a mulher do Sistema de Informação de Agravos de Notificação, com foco nas declarações de óbito (parte III - local de residência, para localizar a família)^(7)^. Já no presente estudo, foi seguida a hierarquia de órgãos públicos estaduais (SSP/AM, DPTC e IML) para consulta da Declaração de Reconhecimento de Óbito (DRO) e da Guia de Liberação de Cadáver, documentos nos quais os familiares foram identificados.

Outro aspecto que merece atenção diz respeito ao treinamento dos pesquisadores envolvidos para abordar e entrevistar esses familiares. Em um estudo realizado em uma metrópole do sudeste brasileiro^(6)^, os pesquisadores foram treinados em autópsia verbal para entrevistar familiares de vítimas de feminicídio. Essa técnica investiga causas e circunstâncias da morte e é realizada logo após o acontecimento. No presente relato de experiência, foi respeitado pelo menos um ano entre o feminicídio e a realização das entrevistas, porque o intuito foi compreender as repercussões do feminicídio na vida das famílias, mediante o oferecimento de espaço para relatarem suas vivências.

Nesse cenário, os pesquisadores podem experimentar uma gama de sentimentos e reações, dada a natureza impactante da temática^(9)^. Em pesquisas qualitativas, o entrevistador não está isento de estabelecer um relacionamento com o participante, e é esperado que o faça com compromisso e ética. Por isso, é necessário que esteja preparado para lidar com a sensibilização de que pode ser tomado, considerando o perfil dos participantes que serão entrevistados, o contexto e o tempo em que estão inseridos e os processos proximais estabelecidos^(2)^.

Pesquisadores que entrevistaram profissionais de saúde e assistência social, que assistem famílias de vítimas de homicídio em sua rotina de trabalho, revelaram dificuldades para realizar as entrevistas, pois houve recusas em participar e em gravar as entrevistas. Incerteza, receio, medo e insegurança foram sensações presentes nos profissionais durante a abordagem com os familiares^(9)^. Essas sensações se aproximaram às vivenciadas pelos pesquisadores do presente estudo ao consultar os documentos dos casos de feminicídio, mas que foram amenizadas com o treinamento realizado no IML antes da realização das entrevistas.

Além da formação e capacitação do pesquisador, Minayo^(1-2)^ propõe critérios para a coleta de dados em pesquisas qualitativas que foram corroborados pelos pesquisadores deste estudo. Esses critérios incluem:

1. Reflexividade: Refere-se às próprias experiências, opiniões, preconceitos e valores dos pesquisadores e como esses aspectos podem influenciar a pesquisa, sobretudo quando a violência é o objeto de investigação;
2. Preparação para o campo: Inclui a revisão da literatura, a definição clara dos objetivos da pesquisa e do plano de trabalho;
3. Ética e sensibilidade cultural: Envolve obter o consentimento informado dos participantes, garantindo a confidencialidade e respeitando as opiniões e valores culturais dos indivíduos entrevistados;
4. Observação: Permite que o pesquisador se adapte e entenda os principais problemas que podem afetar a qualidade dos dados;
5. Registros: De forma cuidadosa e sistemática, documentar o aprendizado e os acontecimentos ao longo do processo de pesquisa;
6. Autoavaliação: Refletir sobre o progresso da pesquisa, as dificuldades e a influência das próprias perspectivas na interpretação dos dados.

Quanto à modalidade das entrevistas, o encontro presencial oferece maior credibilidade e confiabilidade em comparação com as entrevistas online, permitindo a obtenção de informações detalhadas e revelando os aspectos intersubjetivos dos entrevistados^(1-2)^. A carga emocional envolvida é considerável, e os pesquisadores estavam preparados para acolher os participantes, mantendo uma atitude investigativa sem transformar a situação em assistência. Caso fosse detectada a necessidade de atendimento ou intervenção profissional, os serviços apropriados seriam indicados, como descrito no Guia de Serviços Públicos disponibilizado.

A residência tem sido um espaço frequentemente utilizado para tratar de assuntos delicados como o feminicídio^(5-8)^. Mesmo com a preferência de alguns participantes pelo IML, foi observado que os familiares se sentiram confortáveis nos ambientes escolhidos, que eram seguros, privativos e com garantia de sigilo.

O roteiro da entrevista semiestruturada proporcionou um momento de desabafo e reflexão para os familiares. A legitimação dos sentimentos e ideias expressos pelos entrevistados foi essencial para a construção do vínculo com os pesquisadores. Respeitando a vivência e sentimentos dos familiares, quando perceberam uma relação legítima e um espaço seguro para falar, sentiram-se mais confiantes para relatar e colaborar sem medo de julgamento^(10)^.

Legitimar a fala dos familiares envolveu consideração e respeito por suas opiniões, experiências e perspectivas. Outras formas de legitimação utilizadas foram: ouvir atentamente, validar sentimentos e experiências, fazer perguntas de esclarecimento, respeitar a diversidade de perspectivas, evitar julgamentos precipitados, apresentar contrapontos de maneira respeitosa, reconhecer a expertise do participante, mostrar interesse genuíno, apoiar e encorajar a expressão e promover um ambiente inclusivo^(10)^.

Ao se deparar com casos de feminicídio, em especial ao ter acesso a informações, imagens e relatos de familiares tanto sobre a ocorrência em si quanto sobre seus reflexos no núcleo familiar, o pesquisador pode ser invadido por sentimentos e percepções de comoção e compaixão. Como ser humano, lidar ou ser confrontado com um crime dessa natureza, cometido na maioria das vezes por alguém íntimo da vítima, desperta incômodos e repulsa. No presente estudo, isso aconteceu. O cuidado está em não se deixar influenciar ou contaminar por essas vivências e não negá-las, mas sim identificá-las. Portanto, ficar comovido, na experiência dos pesquisadores, acabou facilitando a aproximação com os entrevistados e sua participação.

### Limitações do estudo

Este estudo apresenta algumas limitações que devem ser consideradas. A dificuldade no contato telefônico com familiares, por indisponibilidade ou inexistência de registro, e a recusa em participar por não se sentirem prontos para falar da experiência do luto ou por considerarem a situação resolvida, nos casos em que o autor do crime estava condenado e preso, devem ser levadas em conta. Além disso, a natureza sensível do tema pode ter influenciado a disposição dos participantes em compartilhar experiências profundamente pessoais e traumáticas. Outra limitação importante é a possível influência emocional dos pesquisadores sobre os dados coletados, apesar dos esforços para manter a objetividade. A escassa produção científica sobre o tema impediu um debate mais aprofundado de acordo com a literatura.

### Contribuições para a Área

O percurso trazido neste relato de experiência para entrevistar famílias de vítimas de feminicídio pode proporcionar aos estudantes, docentes, profissionais e pesquisadores da área da saúde, em específico enfermeiros, estratégias que reflitam em suas práticas. Ao desenvolver atividades assistenciais ou científicas dessa natureza, poderão ser terapêuticos e valorizar a família que vivencia as repercussões do feminicídio. A participação em investigações desse tipo permite, também, interseccionar a profissão com outros temas sociais, como violência e gênero.

## CONSIDERAÇÕES FINAIS

A experiência perpassou aspectos teóricos-metodológicos planejados e executados em coleta de dados qualitativos pelos pesquisadores. Ao vivenciarem tal processo, puderam desenvolver habilidades e sensibilização com os casos e nos contatos com os familiares entrevistados.

As reuniões do grupo de pesquisa (GREIVI), o conhecimento obtido na literatura, a experiência dos pesquisadores em entrevistas qualitativas, somada à capacitação recebida pelo IML, foram essenciais para abordar os familiares e falar sobre um assunto tão marcante em suas vidas. Além disso, serviram de base para permitir aos pesquisadores direcionar a ordem de comunicação e utilizar linguagem adequada, tanto nas ligações telefônicas quanto nas entrevistas presenciais.

Realizar pesquisas com famílias de vítimas de feminicídio é um processo desafiador e sensível. O pesquisador precisa demonstrar disponibilidade e respeito para maior interação com o participante, fazendo com que a obtenção dos dados ocorra com qualidade. Para tanto, além de conhecimento e preparo, é esperada uma atitude ética, sem abrir mão da capacidade empática junto à população alvo do estudo. É recomendado, por outro lado, o aperfeiçoamento das políticas públicas de atenção aos familiares de vítimas de feminicídio. Políticas dessa natureza poderiam, com o auxílio da assistência psicológica, social e jurídica, ajudar na elaboração do luto e na superação das consequências da violência vivenciada.
